# Genome-Wide Association Analysis Combined With Quantitative Trait Loci Mapping and Dynamic Transcriptome Unveil the Genetic Control of Seed Oil Content in *Brassica napus* L.

**DOI:** 10.3389/fpls.2022.929197

**Published:** 2022-07-01

**Authors:** Chuanji Zhao, Meili Xie, Longbing Liang, Li Yang, Hongshi Han, Xinrong Qin, Jixian Zhao, Yan Hou, Wendong Dai, Caifu Du, Yang Xiang, Shengyi Liu, Xianqun Huang

**Affiliations:** ^1^Guizhou Rapeseed Institute, Guizhou Academy of Agricultural Sciences, Guiyang, China; ^2^Key Laboratory of Biology and Genetic Improvement of Oil Crops, The Ministry of Agriculture and Rural Affairs, Oil Crops Research Institute, Chinese Academy of Agricultural Sciences, Wuhan, China; ^3^Biosystematics Group, Experimental Plant Sciences, Wageningen University and Research, Wageningen, Netherlands

**Keywords:** *Brassica napus*, seed oil content, distant hybridization, QTL mapping, syntenic gene, GWAS

## Abstract

Rapeseed, an allotetraploid oil crop, provides vegetable oil for human consumption. The growing demand for oilseeds has necessitated the development of rapeseed varieties with improved quality. Therefore, a clear understanding of the genetic basis underlying the seed oil content (SOC) is required. In this study, a natural population comprising 204 diverse accessions and recombinant inbred lines (RILs) derived from *Brassica napus* and *Sinapis alba via* distant hybridization were collected for genome-wide association analysis (GWAS) and quantitative trait loci (QTL) mapping of the SOC trait, respectively. The variable coefficient of the RIL and natural populations ranged from 7.43 to 10.43% and 8.40 to 10.91%. Then, a high-density linkage map was constructed based on whole genome re-sequencing (WGS); the map harbored 2,799 bin markers and covered a total distance of 1,835.21 cM, with an average marker interval of 0.66 cM. The QTLs for SOC on chromosome A07 were stably detected in both single and multiple environments. Finally, a novel locus *qA07.SOC* was identified as the major QTL for SOC based on the GWAS and RIL populations. In addition, the RNA-seq results showed that photosynthesis, lipid biosynthesis proteins, fatty acid metabolism, and unsaturated fatty acid biosynthesis were significantly different between the developed seeds of the two parents of the RIL population. By comparing the variation information and expression levels of the syntenic genes within *qA07.SOC* and its syntenic genomic regions, as well as through haplotype analysis *via* GWAS, *BnaA07.STR18*, *BnaA07.NRT1*, and *BnaA07g12880D* were predicted as candidate genes in the *qA07.SOC* interval. These stable QTLs containing candidate genes and haplotypes can potentially provide a reliable basis for marker-assisted selection in *B. napus* breeding for SOC.

## Introduction

Rapeseed (*Brassica napus* L., 2n = 38, AACC) belongs to the family *Brassicaceae* and is one of the major oilseed crops that provide vegetable oil for human consumption worldwide. With an increase in global human population, improving seed oil content (SOC) has become an important goal in oilseed rape breeding programs (Sun et al., 2018); accordingly, understanding the genetic basis of SOC is necessary to improve the breeding value of rapeseed.

Most agronomic traits of crop plants are simultaneously controlled by multiple genes and influenced by the environment (Zhou et al., 2021). In the case of rapeseed, seed oil- contributing traits are of utmost importance, and they reflect the accumulated effect of a large number of genes expressed throughout the life span of the plant. However, the final plant phenotype is the result of interactions between environmental and genetic factors. Therefore, a genotype × environment interaction is an important aspect to consider when dissecting agronomic traits in *B. napus* (Xie et al., 2020).

Quantitative trait loci (QTL) mapping and genome-wide association analysis (GWAS) are effective strategies to dissect complicated genetic bases, and can help accelerate the breeding of rapeseed by marker-assisted selection. Several studies have reported multiple QTLs for the seed oil-related traits, distributed over all chromosomes in the rapeseed genome (Basunanda et al., 2010; Wang et al., 2010; Wei et al., 2011; Yang et al., 2012; Liu et al., 2015; Guo et al., 2017; Sun et al., 2018; Shi et al., 2019). Similarly, since the release of the *B. napus* reference genome, GWAS of seed oil content has substantially contributed to the related literature. A total of 52,157 SNP markers were obtained in 521 *B. napus* accessions using a 60 K chip array, and 50 QTLs were obtained by GWAS for oil content, of which 29 were newly identified (Liu et al., 2016).

The SOC of *B. napus* is mainly controlled by maternal effects, embryo gene effects, pollen instinct, cytoplasmic effects, and the corresponding gene-environmental interaction effects, which conforms to the additive-dominant-epistatic genetic model and are based on additive and dominant inheritance with high generalized heritability (Wang et al., 2010; Hua et al., 2012; Guo et al., 2017). Oil content has an obvious dynamic trend in the process of seed development and is closely related to multiple biological pathways, such as plant photosynthesis, seed development, material transport, lipid synthesis, accumulation and degradation, all of which form a regulatory network regulated by multiple genes (Liu et al., 2012; Chao et al., 2017; Shi et al., 2017). The biosynthesis of seed oil is divided into two stages: fatty acid synthesis and triacylglycerol synthesis, and is mainly stored as triacylglycerol, which accounts for 60% of the seed mass (Frentzen, 2010). The synthesis of triacylglycerol requires the interaction of many subcellular structures and multiple pathways, and many regulatory and enzyme genes are involved in the whole process (Hills, 2004; Bates et al., 2014). The key enzyme-encoding genes play an important role in the synthesis of rapeseed oil, and its effect on the SOC of *B. napus* mainly depends on the expression of the gene and the regulation the enzyme activity, The genes involved in this process mainly catalyze the synthesis of fatty acid chains and triacylglycerol, including *ACCase*, *Fat A*, *Fat B*, *GPAT*, *LPAAT*, and *DGAT* (Turnham and Northcote, 1983; Weselake et al., 2008; Lock et al., 2009). Problems in the expression or regulation of any kind of enzymes will affect the SOC. Key transcription factors also affect the SOC in *B. napus*, mainly regulating seed development and seed oil accumulation. Previous studies have shown that transcriptional factors, including *WRI1*, *LEC1*, *LEC2*, *FUS3*, and *ABI3* can increase seed oil content by activating or inhibiting the expression of genes related to seed oil synthesis (Kagaya et al., 2005; Wang et al., 2007; Wu et al., 2014; Elahi et al., 2015, 2016). In addition, miRNA may also play an important role in fatty acid and lipid metabolism in the seeds of *B. napus* (Wang et al., 2017). Therefore, a combination of QTL mapping and dynamic transcriptome analysis is an effective strategy for uncovering the genetic basis of the SOC trait in *B. napus*.

In this study, we collected 204 accessions of *B. napus* for GWAS and constructed a recombinant inbred lines (RIL) population of 158 individuals. We used this RIL population to construct a high-density genetic map of 2,799 bin markers, with a total coverage distance of 1,835.21 cM. We evaluated SOC traits across nine different environments for QTL mapping in the RIL population and 3 consecutive years for GWAS. A major QTL on chromosome A07 was detected. Combined dynamic transcriptome, synteny analysis, variant detection, and haplotype analysis were conducted in the major QTL region, and *BnaA07g12790D*, *BnaA07g12830D*, and *BnaA07g12880D* were predicted to be candidate genes. Our findings could contribute to improving the understanding of the genetic basis and breeding of the SOC trait in *B. napus*.

## Materials and Methods

### Plant Materials and Phenotypic Evaluation

A *Sinapis alba* L. inbred line with a high 1,000-seed wight was selected as a male parent to cross with a *B. napus* L. inbred line “*Darmor*.” Well-grown F_1_ lines were obtained by *in vitro* culture of plant embryos for embryo rescue. Using the microspore culture method, GRG2462 was generated in *F*_2:3_ lines with low SOC as female parent. We selected another high-SOC *B. napus* inbred line GRD328 as the male parent, in which the high-oil-content hybrid rapeseed Youyan2020 was developed using GRD328. Finally, a *B. napus* RIL population (*F*_2:11_) consisting of 158 individuals was derived from the crossing of GRG2462 × GRD328, and used for QTL mapping study. A total of 204 *B. napus* inbred lines (Supplementary Table 1) collected worldwide were used for GWAS of the SOC trait. In the field, 120 individuals of each inbred line were planted in a 2.5 × 2.0 m^2^ plot, and the field tests followed a randomized design. The field experiments for QTL mapping in the RIL population were replicated in six locations, namely Guiyang (GY), Qinghe (QH), Changshun (CS), Tangtou (TT), Jinsha (JS), and Badong (BD).

At maturity, the natural population, RIL population, and two parents (GRG2462 and GRD328) were evaluated for SOC in each environment. Foss NIR Systems 5000 Near-Infrared Reflectance Spectroscopy was performed to measure SOC (Tang et al., 2019). SPSS 22 (IBM SPSS, Armonk, NY, United States) was used to calculate the phenotypic variation and frequency distribution.

### Whole-Genome Resequencing, SNP Calling, and Genotyping

All lines in the RIL and GWAS populations were sampled, and genomic DNA was extracted using the Hi-DNAsecure Plant Kit (TIANGEN, Beijing). Genomic DNA was diluted to a final concentration of 20 ng/μl and used to construct a DNA library. The DNA library was subjected to whole-genome resequencing (WGS) using the Illumina NovaSeq 6000 system. Clean reads were obtained by removing adaptor sequences, contamination, and low-quality reads from the raw reads. A Burrows-Wheeler Aligner (BWA; parameter: mem -t 4 -k 32 -M; http://bio-bwa.sourceforge.net/) was used to align the clean reads against the *B. napus* “*Darmor-bzh*” reference genome (https://www.genoscope.cns.fr/brassicanapus/data/; Li and Durbin, 2009; Chalhoub et al., 2014). Alignment duplication was removed using SAMTools (parameter: rmdup; http://samtools.sourceforge.net/; Li et al., 2009). SNP calling within the mapping population was performed by an in-house pipeline in the “Sentieon Genomics” tool (Freed et al., 2017). To exclude false variants, SNPs were filtered by GATK (version 4.1.4.0) based on the following parameters: QUAL < 30.0||MQ < 50.0||QD < 2.0, cluster size 3, and cluster window size 10 (Mckenna et al., 2010). Subsequently, 15 SNPs with a sliding window size and an SNP step size were used to screen the chromosomes. When the number of allelic SNP A:A (or B:B) in the sliding window was less than 11, allelic SNP A:B was used as the genotype to fill and correct this position (Huang et al., 2009).

For subsequent genetic analysis of the RIL population, the recombination of all offspring genotypes in the RIL population was analyzed to divide the bin markers. No recombination events were considered to be present in one Bin marker interval. To ensure the quality of the genetic map, bin markers were filtered according to the following criteria: (1) being less than 10 kb in length; (2) having severe partial separation (chi-square test, ^***^p < 0.001); (3) chromosomes with a small density of bin marker, add some Bin markers with unfixed parameters in terms of length and partial separation. The bin markers that met the criteria were used to construct a high-density genetic map. As per general genetic coding rules, the model “aa × bb” was used to analyze the genotype bin markers in the 158 RILs (Ren et al., 2020).

### Construction of a Genetic Linkage Map

The selected bin markers were assigned to 19 chromosomes in the *B. napus* reference genome according to their genetic position. Each chromosome was considered the corresponding linkage group. The HighMap software (http://highmap.biomarker.com.cn/; Liu et al., 2014) was used to combine co-segregating markers (SNP and/or InDel) into bin markers and estimate the genetic distance between adjacent markers in each linkage group. In the genetic map, centimorgan (cM) distances were calculated to map genetic distances (Kosambi, 2016). To evaluate the quality of the genetic map, we analyzed the pair-wise recombination values of all mapped markers by the Kosambi mapping function of RECORD_WIN (Truco et al., 2013), as well as the collinearity between the genetic map and the *B. napus* reference genome.

### QTL Identification and Analysis

The QTL for SOC in a single environment was identified using the composite interval mapping (CIM) method (Zeng, 1994) in Windows QTL Cartographer software (WinQTLCart, version 2.5; https://brcwebportal.cos.ncsu.edu/qtlcart/WQTLCart.htm; Silva et al., 2012). The logarithm of the odds (LOD) threshold was calculated by a permutation test, with the following parameters: 1.0 cM intervals with a mapping window of 10 cM, five control markers, and a significance level of *p* < 0.05, *n* = 1,000 permutation (Zhang et al., 2019).

Quantitative trait loci × environment interaction effects (QTL by environment interaction in biparental population) were identified by the MET functionality and ICIM-ADD mapping method in the QTL IciMapping V4.1 software (http://www.isbreeding.net/; Li et al., 2008). Determination of the LOD threshold was followed by a significance test (*p* < 0.05) with 1,000 permutations.

### GWAS for SOC

The SOC trait for three consecutive years (2014–2015 CS, 2015–2016 CS, and 2016–2017 CS) was measured, and the best linear unbiased prediction (BLUP) for each accession was obtained using an R script lme4 (CRAN-Package lme4 (r-project.org)) and lsmeans (Lu et al., 2019). The ADMIXTURE software (Version 1.3.0) was used to analyze the population structure in the natural populations (Alexander et al., 2009). The subgroup number (*k*) was set as 1–10, and the probability value (Q) of each material in each subgroup was calculated. The optimal number of subgroups was determined according to the cross-validation error (CV value), and each material was classified into the subgroup to which it belonged according to the *Q* value. The TASSEL 5.0 software was used to calculate the genetic relatedness between two specific accessions and the relative value of genetic relatedness between any accessions, which determined the kinship of the *B. napus* accessions in the natural population (Yu et al., 2006). The PopLDdecay and LDBlockShow softwares were used to analyze the linkage disequilibrium (LD) of the whole genome and the specific region (Zhang et al., 2018; Dong et al., 2020). The Efficient Mixed-Model Association eXpedited (EMMAX) was used to analyze the relative kinship of the natural population. A Mixed Linear Model (MLM) with EMMAX, and Bayesian-information and Linkage-disequilibrium Iteratively Nested Keyway (BLINK) were adopted for association analysis (Huang et al., 2019). The significant *p* value thresholds of the GWAS were set as −log_10_*P* (*p* = 1/total SNPs). The quantile-quantile (Q-Q) plot was shown with the expected *p* value and −log_10_*P* of each SNP, and the Manhattan plot was demonstrated using the R package qqman.

### RNA-Seq and qRT–PCR Analysis

The developed seeds of GRD328 and GRG2462 at 35, 40, 45, and 50 days after flowering (DAF) were collected for RNA extraction and RNA-seq. High-quality RNA was extracted using the RNA Prep Pure Plant Kit (Tiangen, Beijing, China) and sequenced using the Illumina NovaSeq 6000 system. Clean reads were acquired, by filtering the raw reads, and then mapped to the *B. napus* “*Darmor-bzh*” reference genome^1^ using the HISAT2 software (Chalhoub et al., 2014; Kim et al., 2015). The FPKM value was used to estimate gene expression levels (Trapnell et al., 2010). Pearson’s correlation coefficient was calculated using the R package to detect correlations between biological replicates (Leng et al., 2013). The DESeq software was used to test the differentially expressed genes (DEGs) based on the selection criteria |log_2_ (Fold Change) | > 1 and *p*_adj_ < 0.05 (Anders and Huber, 2010). The Kyoto Encyclopedia of Genes and Genomes (KEGG) of DEGs and heatmaps were generated using the OmicShare tools.^2^

Total RNA was reverse transcribed using the HiScript III 1st Strand cDNA synthesis kit (Vazyme, Nanjing, China) following the manufacturer’s instructions. Quantitative real-time PCR (qRT-PCR), with the *BnaActin* gene as an internal control, was performed in a CFX Connect Real-time PCR system (Bio-Rad, United States) using ChamQ™ SYBR qPCR Master Mix (Vazyem, Nanjing, China). Relative gene expression levels were determined using the 2^(-ΔΔCT)^ method (Livak and Schmittgen, 2001). The primers used for qRT-PCR are listed in Supplementary Table 2.

### Candidate Genes Analysis in the Major QTL Interval

The syntenic properties of the genomic regions were used to exclude genes while determining the candidates responsible for QTL-SOC (Zhang et al., 2019). The positional information of SNP and bin markers was used to determine the major QTL interval in the *B. napus* reference genome. The two parents were deeply sequenced on the Illumina NovaSeq 6000 system, and the homozygous polymorphic SNP and InDel between the two parents was used for variant analysis in SnpEff 5.1.^3^ The genes within the major QTL and its syntenic block (or QTL) of another sub-genome in *B. napus* were selected to analyze the unique gene and syntenic gene, along with their variation information and expression levels, based on the deep WGS and RNA-seq data, respectively.

## Results

### Phenotypic Variation of the GWAS and RIL Population

**Figure 1:**
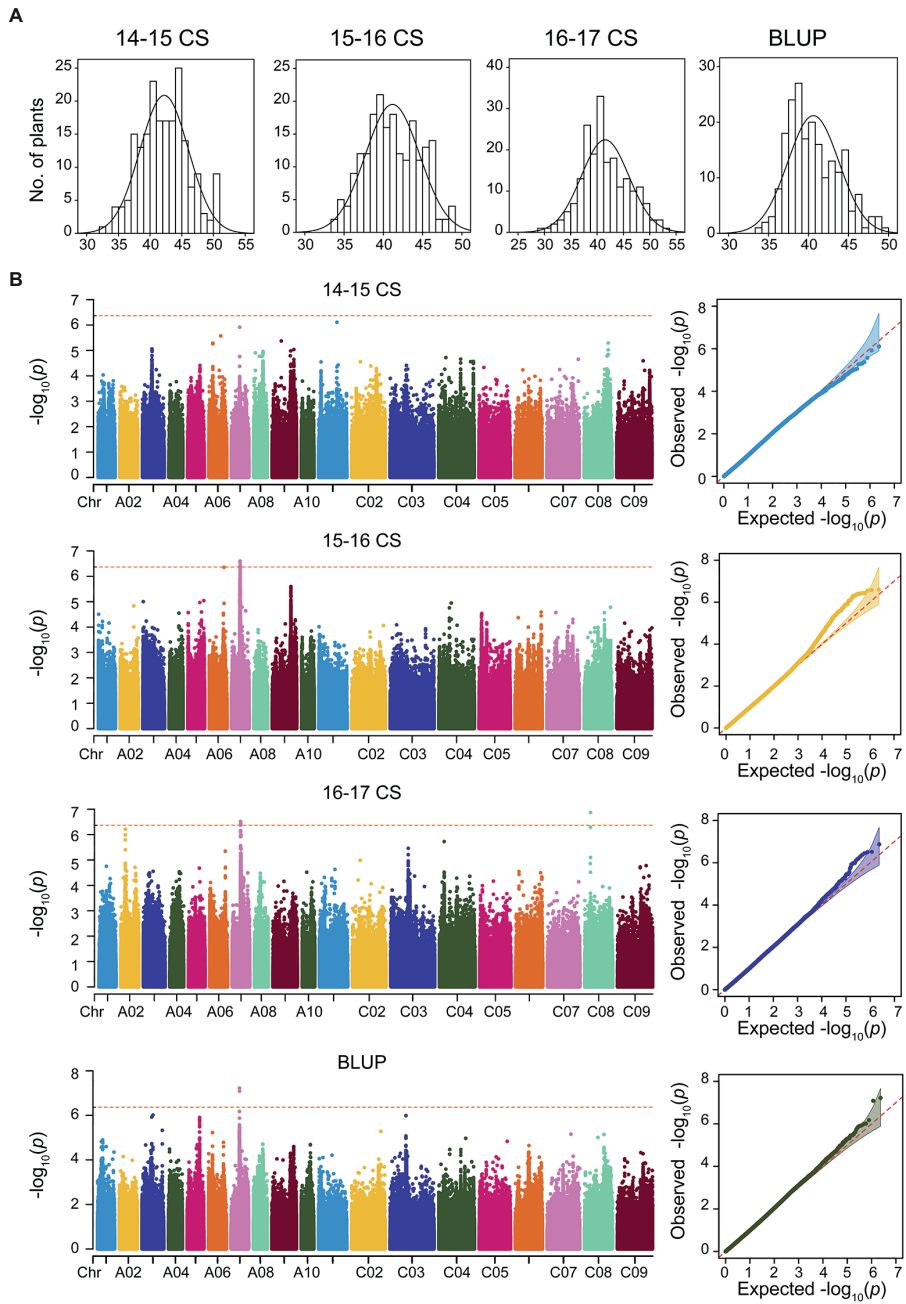
Genome-wide association analysis (GWAS) for the seed oil content (SOC) trait in consecutive 3 years based on Mixed Linear Model (MLM) of Efficient Mixed-Model Association eXpedited (EMMAX). **(A)** The phenotype distributions of SOC in consecutive 3 years and best linear unbiased prediction (BLUP). **(B)** GWAS and Q-Q plot for SOC in consecutive 3 years and BLUP.

Seed oil content showed significant differences between the two parents in each environment (Supplementary Table 3). The frequency distributions of SOC in the RILs in the nine environments displayed normal distributions with considerable transgressive segregation (Supplementary Figure 1). In summary, combined with the SOC trait in nine environments, the minimum SOC was less than 35%, while the maximum was over 41% (Table 1), and the coefficient of variation ranged from 7.43 to 10.91% (Table 1). To test the effects of genotype (G), environment (E), and their interactions (G × E), a variance analysis (ANOVA) was conducted, and significant variation (*p* < 0.01) was observed among environments and genotypes, with the SOC having high narrow sense heritability of 78.54% (Supplementary Table 4). In the natural population, the frequency distributions for SOC in the consecutive 3 years and BLUP displayed normal distributions (Figure 1A). A significant variation in SOC was observed in the natural population; for example, SOC ranged from 33.69 to 49.29%, with an average of 40.60% in BLUP; the coefficient of variation was 7.88% (Table 1). The correlations between environments for association and RIL population were, respectively, less than 75 and 45% (Supplementary Figures 2A,B), suggesting that the phenotypes were greatly influenced by the environments. Overall, these findings indicate that the SOC trait was stably inherited and was suitable for QTL mapping and GWAS.

**Table 1:** Phenotypic variations in the recombinant inbred line (RIL) and natural population.

Population Type	Environment	Min	Max	Mean	*SE*	*SD*	Var	Kurtosis	Skewness	CV (%)
RIL	2012–2013 GY	26.4	45.83	38.78	0.35	4.06	16.52	0.895	−0.883	10.47
	2013–2014 QH	27.93	45.42	36.49	0.27	3.31	10.95	−0.210	0.022	9.07
	2014–2015 CS	34.01	47.74	41.56	0.3	3.29	10.8	−0.592	−0.498	7.91
	2015–2016 GY	25.01	42.1	36.2	0.2	2.69	7.21	1.898	−0.791	7.43
	2015–2016 CS	28.72	41.71	35.53	0.24	2.72	7.4	−0.317	−0.245	7.66
	2016–2017 CS	24.07	48.12	39.84	0.29	3.49	12.2	1.872	−0.589	8.76
	2017–2018 TT	31.06	49.03	38.32	0.25	3.07	9.41	0.611	0.292	8.02
	2017–2018 JS	21.67	46.74	38.87	0.27	3.29	10.84	4.842	−1.237	8.46
	2018–2019 CS	29.94	43.52	37	0.23	2.81	7.89	−0.343	−0.123	7.59
GWAS	2014–2015 CS	32.02	50.77	42.24	0.27	3.82	14.62	−0.316	0.081	9.04
	2015–2016 CS	33.50	49.01	41.17	0.24	3.46	11.96	−0.733	0.092	8.40
	2016–2017 CS	29.24	53.39	41.53	0.32	4.53	20.53	−0.169	0.207	10.91
	BLUP	33.69	49.29	40.60	0.22	3.20	10.26	−0.286	0.547	7.88

Env, environment; Min, minimum value; Max, maximum value; Mean, mean value; SE, standard error; SD, standard deviation; Var, variance; and CV, coefficient of variation.

### Identification of Association Loci for Seed Oil Content

**Figure 2:**
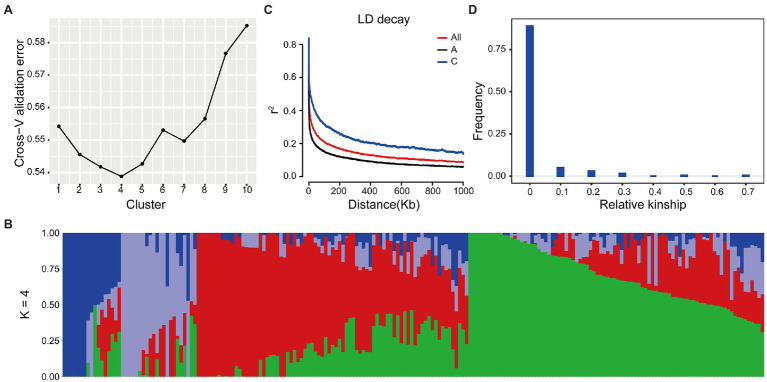
Population structure of *Brassica napus* accessions. **(A)** The value of cross−validation error in different *K* value. **(B)** Model-based group analysis in *K* = 4. The *y* axis quantifies clusters membership, and the *x* axis lists the different *B. napus* accessions. **(C)** Genome-wide and sub-genome average LD decay estimated. **(D)** Relative kinship of 204 *B. napus* accessions.

Based on the WGS data, a total of 2,340,881 SNPs were retained and unevenly distributed on all 19 *B. napus* chromosomes with minor allele frequency (MAF) > 0.05 in the association panel (Supplementary Figure 3; Table 2). The average SNP number is 123,204 on each chromosome (Table 2). According to the Q and K value, we confirmed that the 204 accessions were divided into four groups (Figures 2A,B). The linkage-disequilibrium (LD) distances of the A and C sub-genomes were 38 and 434 kb (*r*^2^ > 0.2), respectively (Figure 2C). In the 204 accessions, the relative kinships among about 89% of the 204 accessions was zero (Figure 2D), suggesting that the genetic relationship between the accessions of natural population was weak and had little influence on further association analysis. According to the results of the MLM with EMMAX, a total of15 SNP loci on chromosomes A07 and C08 associated with SOC were identified at the suggestive threshold excepted 2014–2015 CS environment, and the SNP_11440137 on chromosomes A07 was identified repeatedly in 2016–2017 CS and BLUP (Figure 1B; Table 3). In additional, even if no significant SNPs were detected in 14–15 CS environment, A07_11440137 and A07_11440139 can be identified with -log_10_(*P*) > 5.0 (Table 3). Based on the BLINK model, we identified the significant SNP A07_ 11440137 in 2016_2017 CS, as well as A07_ 11440137 and A07_ 11440139 in BLUP (Supplementary Figure 4; Supplementary Table 5), which suggested that the BLINK results were as same as those of EMMAX in 2016_2017 CS and BLUP. In the BLINK model, the SNPs A07_11440137 and A07_11440139 were also identified with log_10_(*P*) > 5.0 in 2015_2016 CS (Supplementary Table 5).

**Table 2:** The statistics of SNP number on each chromosome.

Chromosome	SNP-number	Chromosome length	SNP/kb
A01	107,089	23,267,856	4.6
A02	105,683	24,793,737	4.26
A03	165,992	29,767,490	5.58
A04	113,531	19,151,660	5.93
A05	147,616	23,067,598	6.4
A06	145,427	24,396,386	5.96
A07	142,496	24,006,521	5.94
A08	89,148	18,961,941	4.7
A09	158,319	33,865,340	4.67
A10	107,570	17,398,227	6.18
C01	128,195	38,829,317	3.3
C02	113,683	46,221,804	2.46
C03	185,302	60,573,394	3.06
C04	141,790	48,930,237	2.9
C05	85,975	43,185,227	1.99
C06	91,498	37,225,952	2.46
C07	129,098	44,770,477	2.88
C08	103,876	38,477,087	2.7
C09	78,593	48,508,220	1.62

**Table 3:** SNP position with significant association for SOC in MLM of EMMAX.

Chromosome	Physical position	*p*-value	Environment
A07	11,440,139	1.21 × 10^−6^	2014_2015 CS
A07	11,440,137	1.74 × 10^−5^	2014_2015 CS
A07	11,343,133	4.21 × 10^−5^	2014_2015 CS
A07	11,411,162	2.68 × 10^−7^	2015_2016 CS
A07	11,630,281	3.85 × 10^−7^	2015_2016 CS
A07	11,630,282	4.16 × 10^−7^	2015_2016 CS
A07	11,630,292	2.82 × 10^−7^	2015_2016 CS
A07	11,632,013	2.52 × 10^−7^	2015_2016 CS
A07	11,632,726	4.04 × 10^−7^	2015_2016 CS
A07	11,774,647	3.67 × 10^−7^	2015_2016 CS
A07	11,778,813	3.71 × 10^−7^	2015_2016 CS
A07	11,428,788	3.57 × 10^−7^	2016_2017 CS
A07	11,428,860	3.07 × 10^−7^	2016_2017 CS
A07	11,440,137	4.26 × 10^−7^	2016_2017 CS
A07	11,575,763	3.16 × 10^−7^	2016_2017 CS
C08	7,894,416	1.35 × 10^−7^	2016_2017 CS
A07	11,440,137	6.02 × 10^−7^	BLUP
A07	11,440,139	8.21 × 10^−7^	BLUP

### Construction of a *Brassica napus* High-Density Linkage Map Based on Whole-Genome Resequencing

For linkage map construction, 158 RILs were performed for whole genome re-sequencing, and a total of 617,466 SNP markers were obtained to constitute 38,516 bin markers (Supplementary Table 6). Subsequently, with division and filtration, 2,799 Bin markers harboring 184,183 SNPs were successfully selected to genotype the 158 RILs (Supplementary Figure 5; Supplementary Table 7).

**Figure 3:**
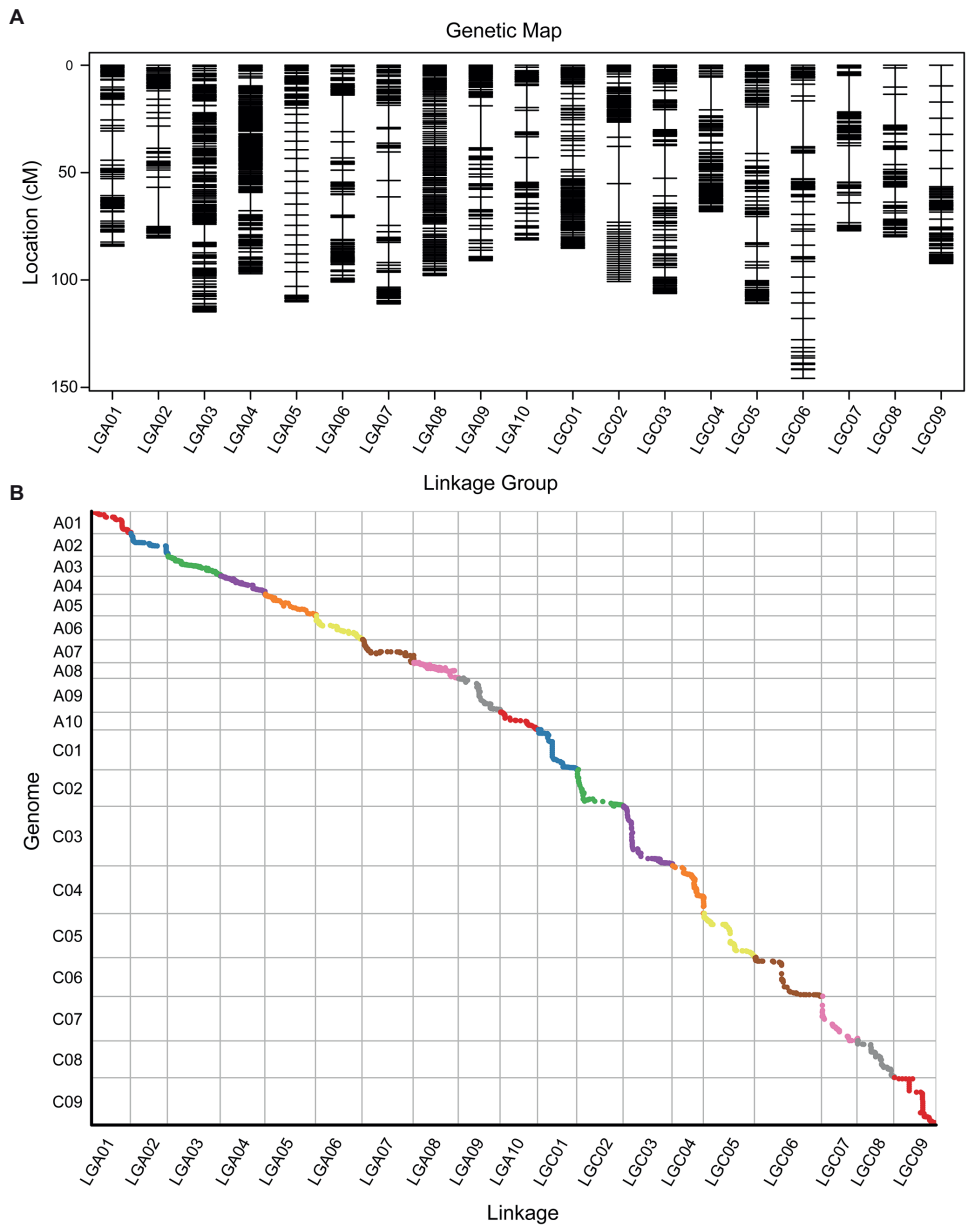
High-density genetic map illustration and collinearity analysis between the genetic map and the genome. **(A)** Distribution of Bin markers on 19 linkage groups. Horizontal black lines on each linkage group represent mapped Bin markers. Vertical lines of each linkage group represent the total genetic distance (cM) of the linkage group. The linkage group ID is shown on the *x*-axis, and the genetic distance is shown on the *y*-axis. **(B)** The *x*-axis is the genetic distance of each linkage group. The *y*-axis is the physical distance of each chromosome, with the collinearity of genomic markers and genetic maps represented in scatter. Different colors represent different chromosomes or linkage groups.

Based on the effective SNPs and bin markers, the map harbored 2,799 mapped bin markers, spanning a total distance of 1,835.21 cM with an average distance of 0.66 cM between adjacent markers (Figure 3A; Table 4). The A-sub-genome harbored 1,577 mapped bin markers with a total distance of 968.89 cM whereas the C-sub-genome harbored 1,222 mapped bin markers with a total distance of 866.32 cM (Figure 3A; Table 4). Among the 19 linkage groups (LG), the longest one was LG C06, reaching 145.79 cM with an average interval distance of 0.87 cM, and the shortest one was LG C04 at only 68.15 cM with an average interval distance of 0.59 cM (Figure 3A; Table 4). The linkage group with the largest number of mapped bin markers was LG A03, which harbored 240 markers, and the linkage group with the least number of bins was LG C09, with only 99 bin markers (Table 4). In the whole genetic map, about 97.35% of the gaps were less than 5 cM, and the max gap of each linkage group ranged from 2.07 to 21.7 cM (Table 4).

**Table 4:** Characteristics of the high-density genetic map.

Linkage group	Total bin marker	Total distance (cM)	Average distance (cM)	Gaps < 5 cM	Max Gap (cM)
A01	128	84.23	0.66	97.64%	13.61
A02	178	80.41	0.45	98.87%	18.27
A03	240	114.82	0.48	99.58%	8.48
A04	231	97.09	0.42	99.57%	8.49
A05	103	110.12	1.07	95.10%	6.81
A06	140	100.92	0.72	96.40%	17.02
A07	137	111.1	0.81	94.85%	13.28
A08	127	97.91	0.77	100.00%	2.07
A09	173	90.96	0.53	98.26%	19.59
A10	120	81.33	0.68	96.64%	11.57
C01	155	85.23	0.55	99.35%	6.38
C02	125	100.7	0.81	97.58%	17.97
C03	122	106.29	0.87	97.52%	15.23
C04	115	68.15	0.59	99.12%	15.28
C05	169	110.94	0.66	97.62%	21.7
C06	167	145.79	0.87	93.98%	21.34
C07	131	77.07	0.59	97.69%	17.02
C08	139	79.85	0.57	97.10%	14.41
C09	99	92.3	0.93	92.86%	9.62
Total	2,799	1,835.21	0.66	97.35%	

The collinearity analysis of the genetic map and physical map of the *B. napus* reference genome showed that the mapped bin markers were consistent with the *B. napus* genome (Figure 3B; Supplementary Tables 8, 9). The average value of Spearman’s correlation coefficient was 99.15%, which suggested that each linkage group had high collinearity with the corresponding chromosome (Supplementary Table 10). Collinearity analysis indicated that the genetic map was exhibiting a high quality.

### QTL Mapping for SOC in the RIL Population

**Figure 4:**
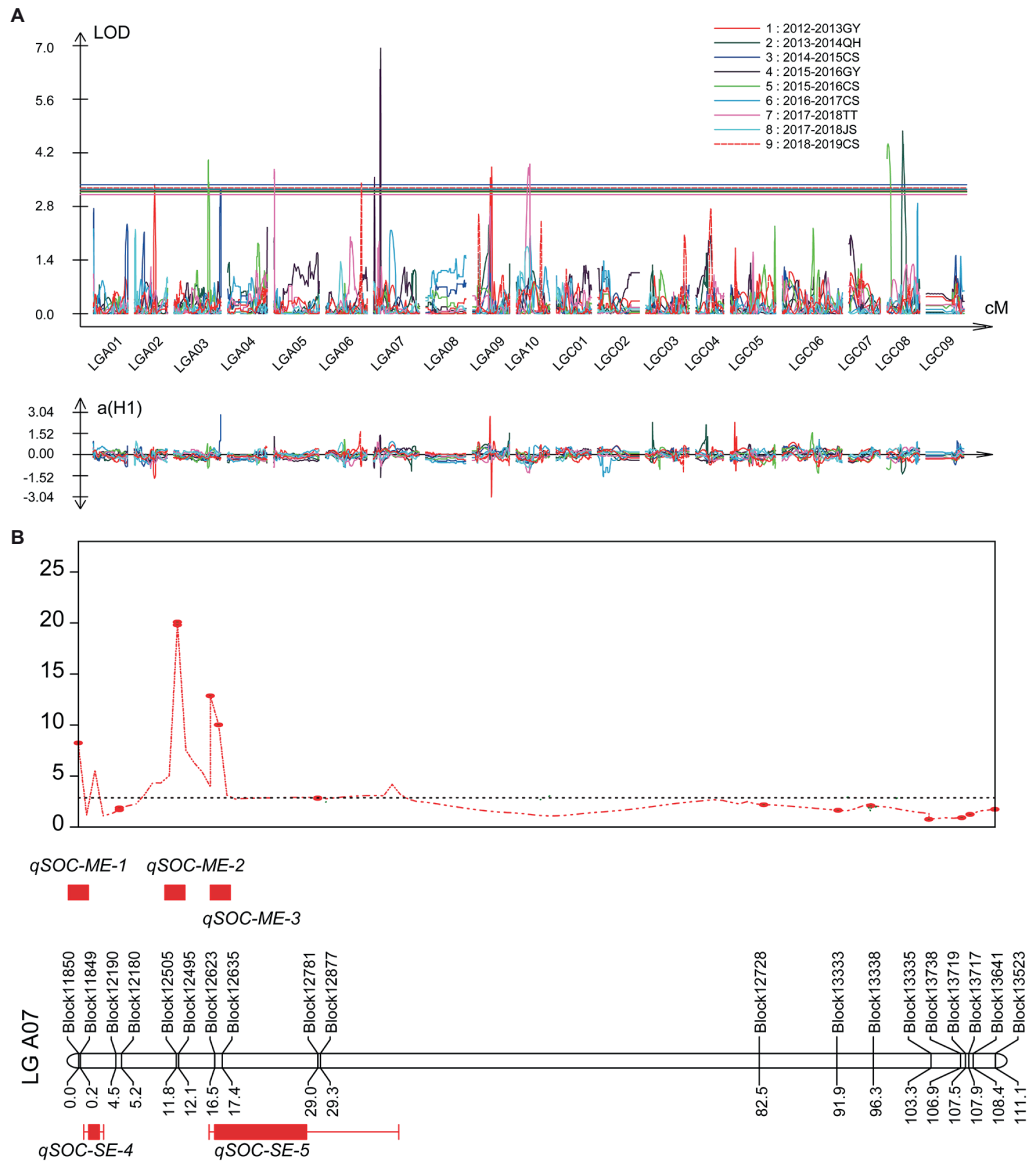
Quantitative trait loci (QTLs) identification for SOC in single and multi-environments. **(A)** The various colors represented the QTL identified in different single environments. **(B)** Co-localization analysis of the major QTLs identified in single and multi-environments on linkage group A07. ME represented multi-environments; SE represented single environment.

In the single environment, a total of nine QTLs with the PVE ranging from 7.97 to 15.07% were identified on chromosomes A02, A03, A05, A06, A07, A09, and C08 in six environments (Figure 4A; Table 5). Among these QTLs, both *qSOC-SE-5* with the highest LOD value of 6.9 and *qSOC-SE-4* with the highest PVE of 15.07% were identified on chromosome A07 (Table 5). In multiple environments, a total of 12 QTLs were detected using QTL IciMapping, and all these QTLs demonstrated a PVE range of 1.08–3.74% (Figure 4B; Supplementary Table 11). Among the 12 SOC QTLs, only *qSOC-ME-2*, *qSOC-ME-3*, *qSOC-ME-5*, *qSOC-ME-6*, and *qSOC-ME-8* were obtained from the high-seed oil parental line GRD328 (Supplementary Table 11). Considering PVE and the environmental factor, we concluded that *qSOC-ME-3* was the major QTL for the SOC trait, named *qA07.SOC*.

**Table 5:** Major QTLs for SOC identified in single environment.

QTL	Chr	Position (cM)	LOD	Interval (cM)	Add	PVE (%)	Environment
*qSOC-SE-1*	A02	48.8	3.4	46.8–51.9	−1.7007	8.49	2012–2013 GY
*qSOC-SE-2*	A09	46.2	3.8	46.1–47.6	−3.0434	9.72	2012–2013 GY
*qSOC-SE-3*	C08	38.2	4.7	37.4–44	−1.3912	11.23	2013–2014 QH
*qSOC-SE-4*	A07	2.1	3.6	0.6–3	−0.9701	15.07	2015–2016 GY
*qSOC-SE-5*	A07	16.5	6.9	15.8–38.8	1.404	11.72	2015–2016 GY
*qSOC-SE-6*	A03	83.8	4	82.4–86.7	−0.9741	11.07	2015–2016 CS
*qSOC-SE-7*	C08	2.3	4.4	0–9.5	−1.0376	13.79	2015–2016 CS
*qSOC-SE-8*	A05	0.3	3.7	0–1.3	−0.9162	8.11	2017–2018 TT
*qSOC-SE-9*	A06	86.5	3.4	85.3–86.9	−0.8491	7.97	2018–2019 CS

SOC, seed oil content; Chr, Chromosome; LOD, logarithm of the odds; PVE, phenotypic variation explained; and Add, additive effect. QTL was named by the consensus QTL nomenclature, and SE represented single environment.

The major QTL *qA07.SOC* linked with the bin markers Block12623 and Block12635 was identified in the single environment of 2015–2016 GY (*qSOC-SE-5*; Figure 4; Table 5). Then, we divided the two groups (AA genotype and BB genotype) based on the genotypes of the twoflanking bin markers (Supplementary Figure 6); for example, the AA genotype represented the genotype of both Block12623 and Block12635, which were consistent with high-seed oil parental line GRD328. In the 158 RIL lines, the bin markers Block12623 and Block12635 divided the AA and BB groups with different significance in the SOC trait (Supplementary Figure 6), which suggested that the major and stable QTL-*qA07.SOC* was reliable for the marker-assisted selection of the SOC trait, even if other minor QTLs are present.

### Global Transcriptome Analysis in the Developed Seeds

**Figure 5:**
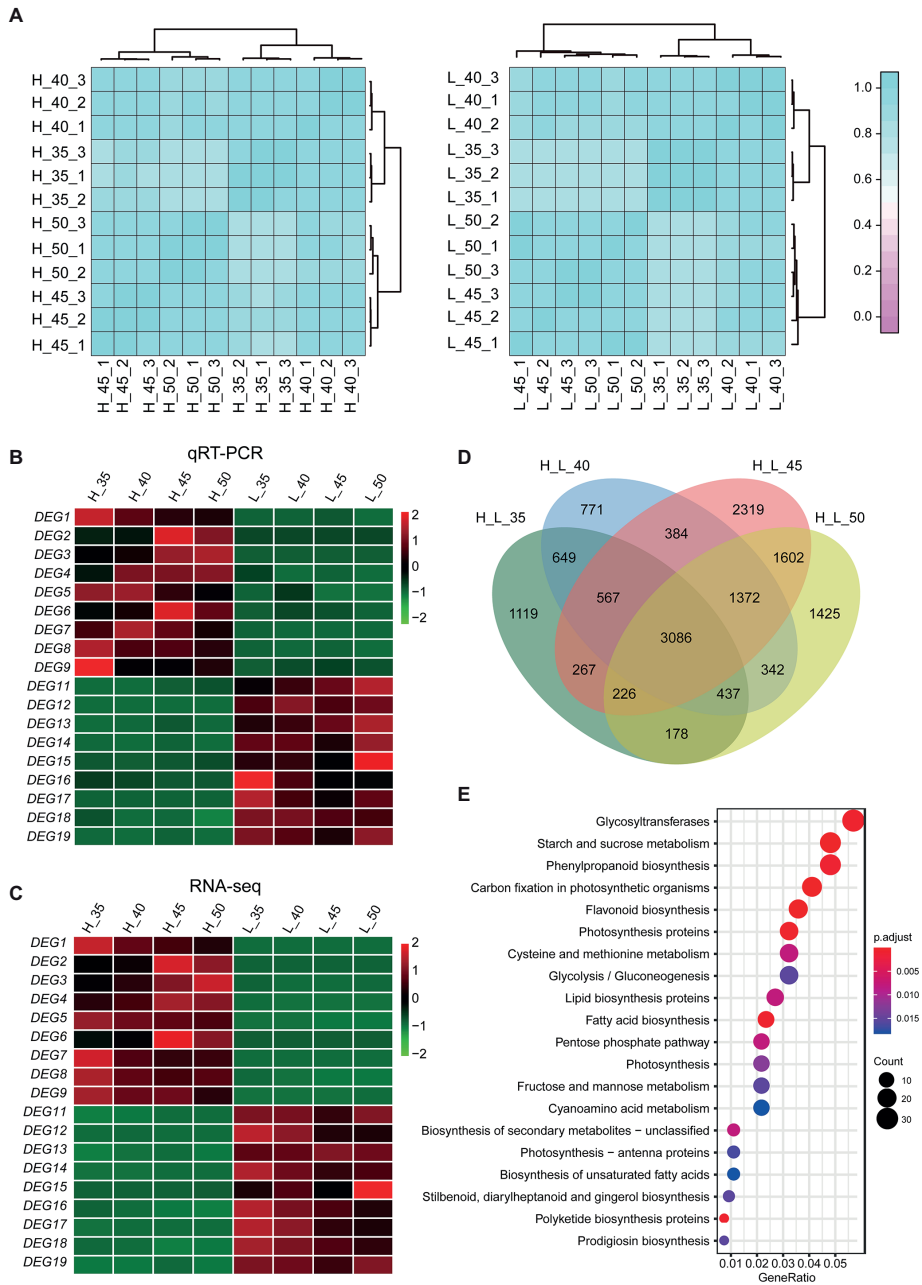
RNA-seq analysis and Kyoto Encyclopedia of Genes and Genomes (KEGG) enrichment analysis of differentially expressed genes (DEGs). **(A)** Spearman correlation plot in developed seeds of GRD328 (high seed oil content) and GRG2462 (low seed oil content). **(B,C)** Heatmap represented that the expression levels of the DEGs based on the RNA-seq was verified by quantitative real-time PCR (qRT-PCR). DEGs 1–9 were upregulated in GRD328, and DEGs 11–19 were downregulated in GRD328. The bars represented the normalized transformed counts of qRT-PCR values **(B)** and FPKM **(C)**, respectively. **(D)** The statistical analysis of DEGs in developed seeds between two parents. **(E)** KEGG enrichment analysis of DEGs.

To study the DEGs and metabolic pathways contributing to the difference in SOC of the two parents, three biological replicates of the developed seeds, at 35, 40, 45, and 50 DAF, were collected for RNA sequencing. A total of 186.99 Gb clean data were obtained, and the average Q30 value was 94.84% (Supplementary Table 12). Spearman correlation based on the FPKM values in samples with high and low oil contents showed good consistency in biological replicates (Figure 5A). In addition, 18 genes differentially expressed between high- and low-oil content samples based on the RNA-seq data were verified by qRT-PCR (Figures 5B,C); results suggesting that the transcriptome data were reliable and could be used for subsequent analyses.

Using the DEseq2 method, 3,086 genes were found to be differentially expressed between the two parents (Figure 5D). To discern the functional distribution of the 3,086 DEGs, KEGG pathway analysis was performed, which showed that the most significantly enriched pathways of the DEGs in the developed seeds were involved in lipid biosynthesis, fatty acid metabolism, unsaturated fatty acids biosynthesis, and photosynthesis (Figure 5E).

### Prediction of Candidate Genes and Mining of Favorable Allelic Variants in the *qA07.SOC* Region

**Figure 6:**
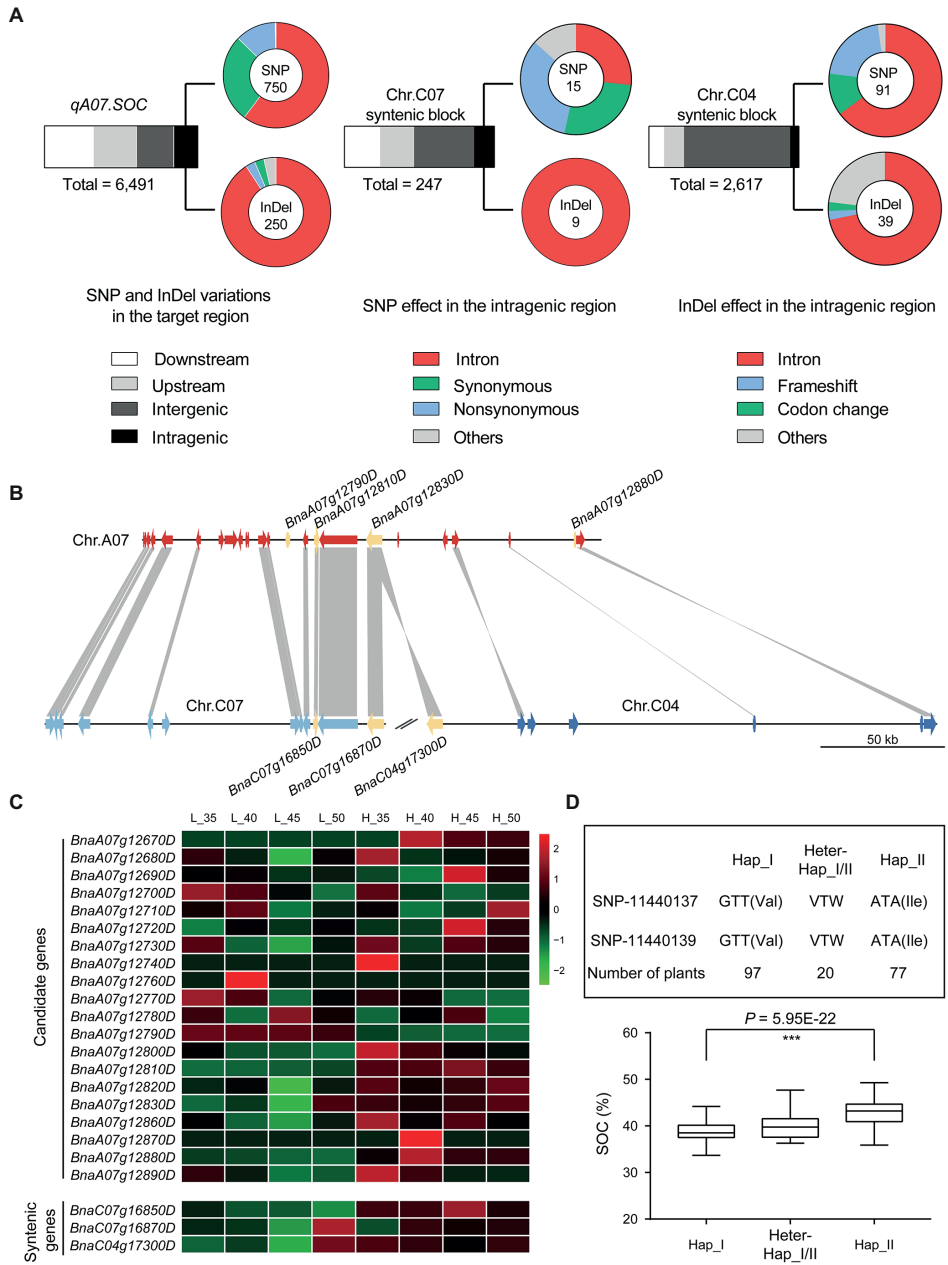
The candidate genes prediction by comparative analysis. **(A)** The variations were identified in *qA07.SOC* and the syntenic regions. **(B)** The syntenic analysis for *qA07.SOC*. **(C)** Heatmap displays the expression levels of the candidate genes and the syntenic genes. The bars represented FPKM normalized transformed counts. **(D)** Haplotype analysis based on GWAS in BLUP.

The *qA07.SOC* region was linked with markers Block12623 and Block12635, which covered 180 kb and consisted of 24 genes, of which eight genes (*BnaA07g12660D*, *BnaA07g12670D*, *BnaA07g12740D*, *BnaA07g12750D*, *BnaA07g12760D*, *BnaA07g12840D*, *BnaA07g12850D*, and *BnaA07g12870D*) were not expressed in the developed seeds of the two parents (Figure 6C; Supplementary Table 13). Four genes, *BnaA07g12790D*, *BnaA07g12810D*, *BnaA07g12830D*, and *BnaA07g12880D*, were differentially expressed between the two parents (Figure 6C; Supplementary Table 13). In DNA levels, the SNP and InDel variations between the two parents in the *qA07.SOC* were analyzed (Figure 6A), and most of the variations were identified in the upstream and downstream of the candidate genes, and only one missense mutation in the coding sequence of *BnaA07g12790D* (Supplementary Tables 14, 15).

According to the syntenic analysis, the syntenic blocks of *qA07.SOC* ranged from 22,766,185-22,905,060 bp on chromosome C07 to 15,347,512-15,556,735 bp on chromosome C04 (Figure 6B). Based on the QTL mapping results in the RIL population, no QTLs for SOC were identified in the syntenic blocks (Figure 4A). After confirming the syntenic block regions, the SNP and InDel variations between the two parents in the syntenic blocks were also analyzed (Figure 6A). In the syntenic block regions, there was no syntenic gene for *BnaA07g12790D* and *BnaA07g12880D* (Figure 6B). The syntenic gene of *BnaA07g12810D* was *BnaC07g16850D*, while both *BnaC07g16870D* and *BnaC04g17300D* were syntenic genes of *BnaA07g12830D* (Figure 6B). As there were no QTLs for SOC in the syntenic blocks, the unique genes *BnaA07g12790D* and *BnaA07g12880D* in *qA07.SOC* could not be excluded. Based on the RNA-seq data, the expression pattern of *BnaA07g12810D* and its syntenic gene *BnaC07g16850D* were coincident in the two parents (Figure 6C; Supplementary Table 16), as well as no variation in the coding sequence of *BnaA07g12810D*, suggesting that *BnaA07g12810D* may be excluded from the *qA07.SOC* region. Consequently, *BnaA07g12790D* (*BnaA07.STR18*) and *BnaA07g12830D* (*BnaA07.NRT1*), which encode a thiosulfate sulfurtransferase and major facilitator superfamily protein, respectively, as well as the unknown functional gene *BnaA07g12880D* were the candidate genes.

According to the results of linkage and association analyses, the major QTL primarily responsible for SOC was located on chromosome A07 (Figures 1, 4). While the mining of favorable allelic variants in the *qA07.SOC*, haplotype analysis in BLUP showed that the SOC phenotype with a significant difference was divided by haplotype I and haplotype II derived from SNP_11440137 to SNP_11440139, respectively (Figure 6D). The favorable allelic variants of SNP_11440137 (A) and SNP_11440139 (A) caused the amino acid to change from valine to isoleucine (Figure 6D). Overall, in this study, these stable QTLs containing candidate genes and haplotypes provided a reliable basis for marker-assisted selection in *B. napus* breeding for SOC.

## Discussion

### Utility of a High-Density Genetic Map in the *F*_2:11_ RIL Population

Most of the complex agronomic traits in *B. napus* are quantitative. Therefore, it is necessary to establish a genetic linkage map with a high accuracy and saturation for QTL mapping. In the early stages of genetic mapping, the stability and reliability of RFLP and SSR markers are relatively high and have been used to study the genetic linkage map of *B. napus* (Landry et al., 1991; Uzunova et al., 1995; Wang et al., 2013; Qi et al., 2014). With the announcement of the *B. napus* reference genome and the high-density of SNP in the genome, the SNP Chip Array became popular in plant research. A *B. napus* linkage map containing 2,771 bins with an average distance of 1.47 cM between bins was constructed for *Sclerotinia* resistance and flowering time QTL mapping based on a 60 K SNP Chip Array (Zhang et al., 2019). In recent years, simplified genome sequencing techniques, such as restriction site-associated DNA sequencing (RAD-seq), have been used to construct linkage maps with a total length of 1,610.4 cM in *B. napus* (Chen et al., 2017).

Previous studies have reported that the number of recombinants with close linkage markers in the RIL population is twice that in the F_2_ population and that the genetic distance of linkage genes could be accurately estimated (Burr et al., 1988). In our study, after the introduction of *S. alba* L. inbred line, an F_2:11_ RIL population was constructed. Then, we used the whole-genome resequencing method to genotype the RIL population and got a high-density genetic map that harbored 2,799 bin markers and covered a total distance of 1,835.21 cM (Figure 3; Table 4), which provided a super foundation for QTL mapping.

### The Co-localization of the Major Locus for SOC by QTL Mapping and GWAS

There are many factors that affect the results of QTL mapping, such as the phenotype heredity, the type and size of mapping population, and the type and density of mapping markers. QTL analysis is carried out on the basis of phenotypic analysis, and different traits have different heritability. Traits with lower heritability are greatly influenced by environment, and changes in environment will lead to changes in the corresponding relationship between genotype and phenotype, and then generate different QTL mapping results. In our study, the mapping population and markers were high quality, while the environment factor mainly effected the result of QTL mapping. In the nine environments, the correlations of the phenotypes were lower than 45% (Supplementary Figure 2B); therefore, the major QTL identified was different among each environment due to the environment factor. However, combined the single and multiple environments, we identified the major QTL was *qA07.SOC*. Similarly, the correlations of the association population were about 55–75% (Supplementary Figure 2A), suggesting that the environment factor also affected the GWAS results. Therefore, we compared the GWAS results between the EMMAX and BLINK, suggesting the same significant SNPs, A07_11440137 and A07_11440139 were repeatedly identified in different environments by two software (Table 3; Supplementary Table 5). Finally, although the environment factor affected the phenotypes of association and RIL populations, the major QTL for SOC was reliable based on the multiple environments and methods.

### Comparison Between Candidate Region With Syntenic Region to Predict Candidate Genes in Allotetraploid *Brassica napus*

Comparison between the syntenic block regions in the different genomes of closely related species can help to predict candidate genes The major QTL-*qSS.C9* for seeds number per silique in *B. napus* was fined mapped in a 50 kb region on chromosome C09. By comparison of the syntenic blocks in chromosome A10 of *B. rapa*, C09 of *B. oleracea* with the candidate region of *B. napus*, the deletion of *BnaC09g45890D* encoding the telomerase-activating protein resulted in a significant reduction in the number of seeds per silique (Li et al., 2015). For allotetraploid *B. napus*, the regions of the syntenic blocks between different sub-genomes can also be compared. In allotetraploid, syntenic blocks with syntenic QTLs may indicate the presence of syntenic genes with conserved functions, although their contributions to the corresponding phenotype may be different (Zhang et al., 2019). Regarding the absence of syntenic QTLs in the syntenic blocks, the unique gene in the major QTL region and the genes with expression patterns different from those of syntenic genes, as well as the genes with unique variations in the major QTL, were retained as candidate genes for further analysis. In our study, according to the expression levels in developed seeds, four genes, including *BnaA07g12790D* (*BnaA07.STR18*), *BnaA07g12810D*, *BnaA07g12830D*, and *BnaA07g12880D* were selected (Figure 6C). In addition, based on the syntenic properties of the genomic regions, *BnaA07g12810D* in the *qA07.SOC* region could be excluded. According to haplotype analysis, the favorable allelic variants located in *BnaA07.STR18* resulted in significant differences in phenotypic variation (Figure 6D). In addition, in *Arabidopsis thaliana*, *AT5G66170* is the orthologous gene of *BnaA07.STR18*, and the Gene Ontology biological process of *AT5G66170* is involved in homeostatic processes, lipid metabolic processes, monocarboxylic acid metabolic processes, pigment biosynthetic processes, and responses to inorganic substance.^4^ Taken together, *BnaA07.STR18* may be a candidate gene conferring high SOC to *qA07.SOC*. These methods and principles will help elucidate polyploid genomics in future studies and provide useful information for *B. napus* breeding programs focusing in SOC.

### *qSOC.A07* Is Available for High-Yield and High-Oil Pyramid Breeding in *Brassica napus*

With an increase in the global human population and growing demand for oil, high yield and high oil content have become important goals in oilseed rape breeding programs (Sun et al., 2018). Thousand-seed weight (TSW) is the major factor affecting rapeseed yield. TSW and SOC are quantitative traits that are controlled by multiple genes and influenced by the environment. Therefore, aggregation of multiple QTLs for TSW and SOC is an important process and method to achieve high yield and high oil content in *B. napus*. Many QTLs for the SOC trait have been identified in previous studies, and a total of 59 QTLs can be mapped onto the *B. napus* reference genome (Zhao et al., 2005; Delourme et al., 2006; Qiu et al., 2006; Sun et al., 2012; Wang et al., 2013; Liu et al., 2016; Zou et al., 2016). While integrating these mapped QTLs to the *B. napus* reference, we found that there were fewer QTLs for SOC on chromosome A07 (Supplementary Figure 7; Fan et al., 2010; Shi et al., 2011; Wang et al., 2016; Zhao et al., 2016). In this study, the major QTL for SOC was located at 180 kb (11,378,925–11,559,658 bp; Figure 4; Supplementary Table 11). The major QTL *qSOC.A07* was found to be novel based on the integration of mapped QTLs (Supplementary Figure 7). In previous studies, QTLs for TSW identified on chromosome A07 overlapped with the region of *qSOC.A07* (Supplementary Figure 7), suggesting that this genomic region covering the loci for TSW and SOC can be used for pyramid breeding. The parent line GRG2462, with high TSW and low SOC, was derived from *B. napus* and *S. alba* by distant hybridization. The major QTL for SOC was identified based on the 158 RILs; using this information, and we hope to breed a new rapeseed germplasm with high yield and high oil content among the RILs in the future.

## Data Availability Statement

The datasets presented in this study can be found in online repositories. The raw data generated in this study have been deposited into the CNGB Sequence Archive (CNSA) of China National GeneBank DataBase (CNGBdb; https://db.cngb.org/) with accession numbers CNP0002896 and CNP0002897.

## Author Contributions

YX, SL, CZ, and MX designed the research. CZ, MX, LL, LY, HH, XQ, JZ, YH, WD, CD, and YX performed the experiments. CZ, MX, LL, LY, and YX analyzed the data. XH provided the materials. CZ, MX, and YX wrote and revised the manuscript. All authors contributed to the article and approved the submitted version.

## Funding

This research was funded by National Natural Science Foundation of China (32070217), Subsidy project from NSFC of Guizhou Academy of Agricultural Sciences [No. (2021)50], and the Agricultural Scientific and Technological Research Projects of Guizhou province [Nos. (2020)1Y106 and 1Y109].

## Conflict of Interest

The authors declare that the research was conducted in the absence of any commercial or financial relationships that could be construed as a potential conflict of interest.

## Publisher’s Note

All claims expressed in this article are solely those of the authors and do not necessarily represent those of their affiliated organizations, or those of the publisher, the editors and the reviewers. Any product that may be evaluated in this article, or claim that may be made by its manufacturer, is not guaranteed or endorsed by the publisher.

## Footnotes

^1^ https://www.genoscope.cns.fr/brassicanapus/data/

^2^ https://www.omicshare.com/tools/

^3^ https://pcingola.github.io/SnpEff/

^4^ https://www.arabidopsis.org/

## Supplementary Material

Supplementary Figure 1 The frequency distributions of the SOC trait in the RIL population.

Supplementary Figure 2 The correlations between environments for the association and RIL population. **(A)** The correlations for the association population. **(B)** The correlations for the RIL population.

Supplementary Figure 3 The density of SNP covered in 19 *Brassica napus* chromosome for GWAS.

Supplementary Figure 4 GWAS for the SOC trait in consecutive 3 years based on BLINK.

Supplementary Figure 5 Genotype map based on the Bin markers of the total 158 RILs.

Supplementary Figure 6 The effect analysis of the major QTL for SOC in 2015–2016 GY. AA and BB was the genotype of each flanking markers linked the major QTL in the two parents. The ‘*n*’ represented the number lines in the RIL population conferring the corresponding genotype. *** represented significance level of the Student’s *t*-test, *p* < 0.001.

Supplementary Figure 7 QTLs for TSW and SOC identification in previous studies.
