# Supplementary figures and images for: Genome-Wide Association Analysis Combined With Quantitative Trait Loci Mapping and Dynamic Transcriptome Unveil the Genetic Control of Seed Oil Content in Brassica napus L
Source: Front Plant Sci. 2022 Jul 1;13:929197. doi: 10.3389/fpls.2022.929197 (PMC9283957; doi:10.3389/fpls.2022.929197)

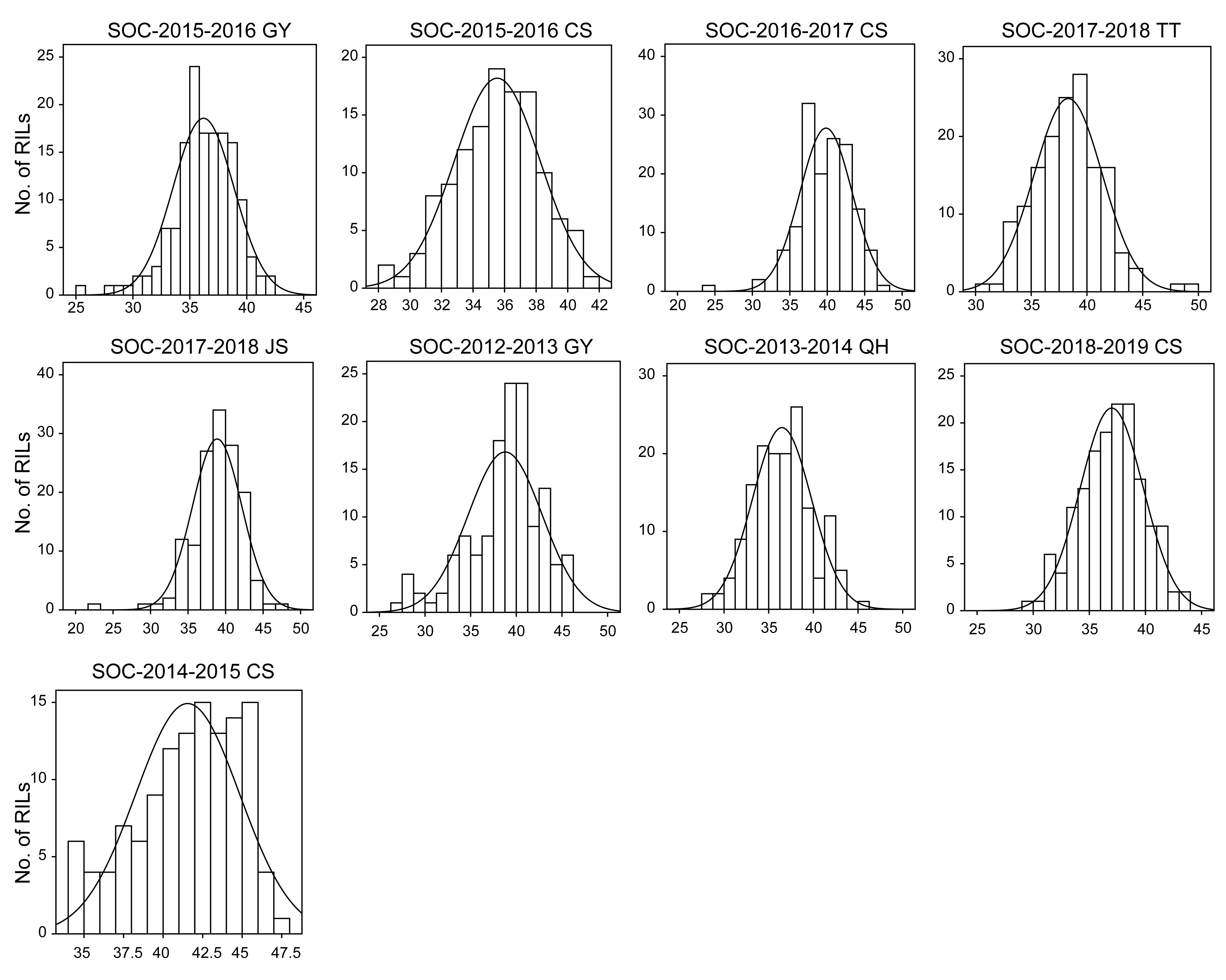

Supplement: Supplementary Figure 1 — The frequency distributions of the SOC trait in the RIL population. [file Image_1.TIF]

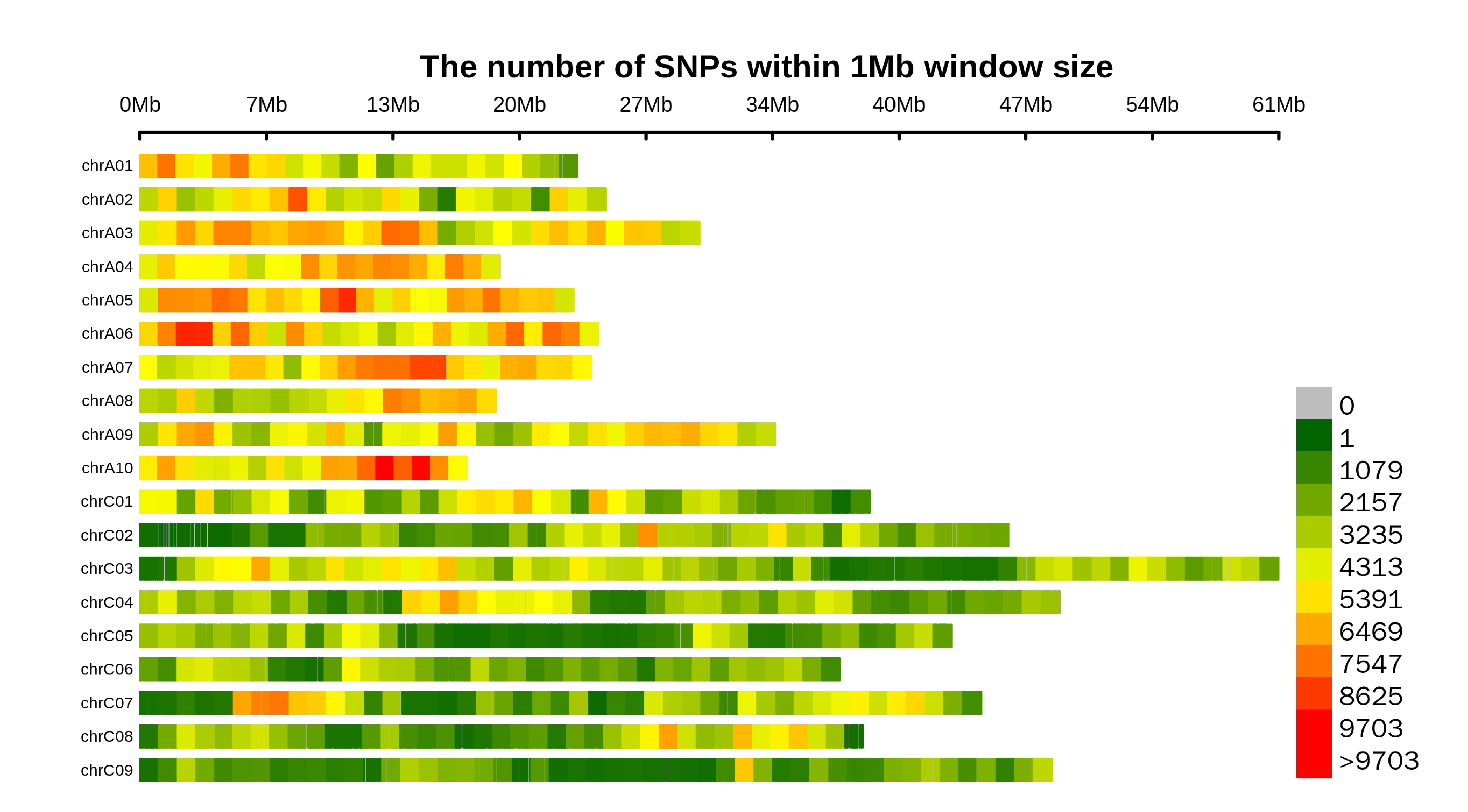

Supplement: Supplementary Figure 3 — The density of SNP covered in 19 Brassica napus chromosome for GWAS. [file Image_3.TIF]

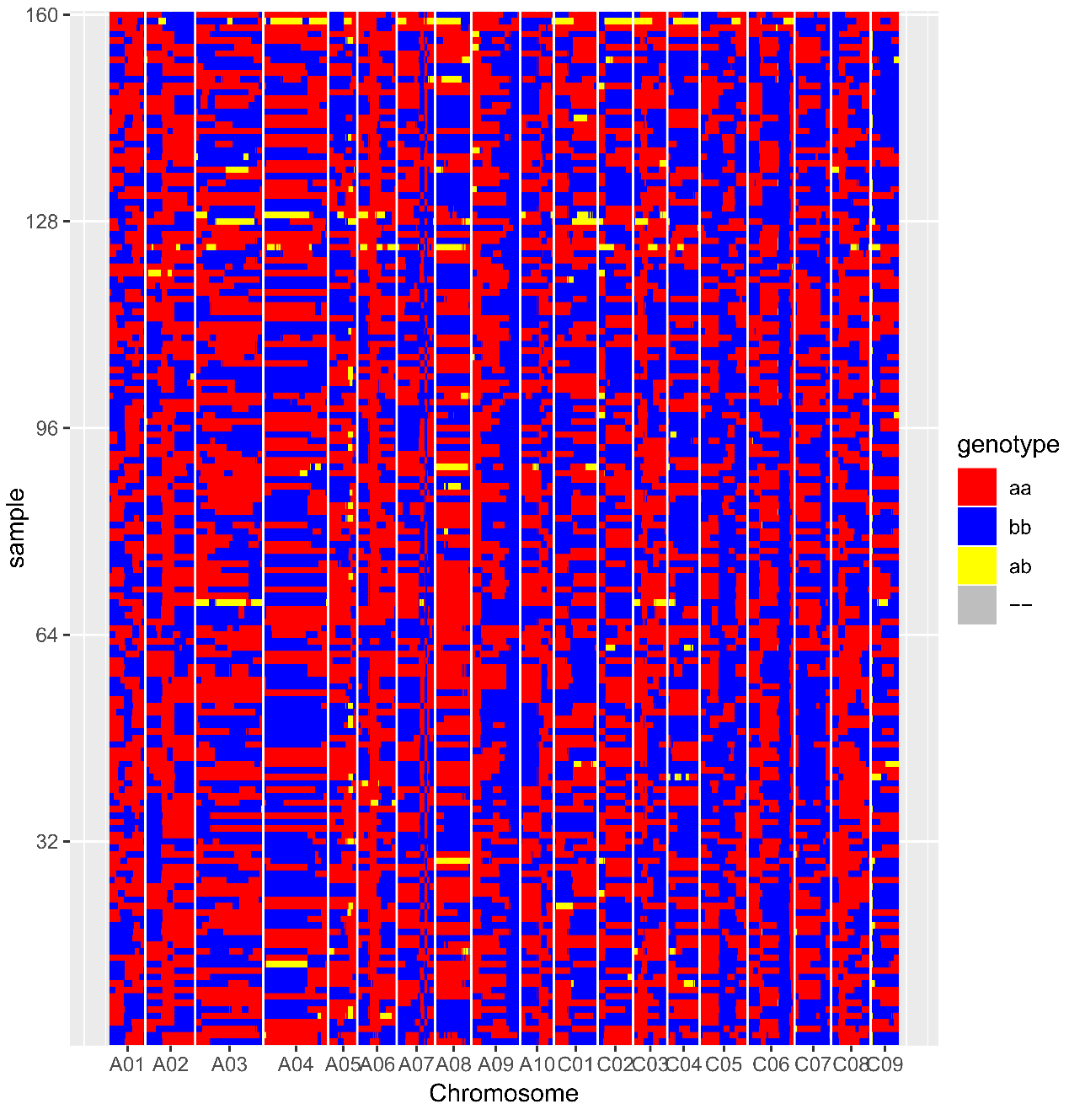

Supplement: Supplementary Figure 5 — Genotype map based on the Bin markers of the total 158 RILs. [file Image_5.TIF]

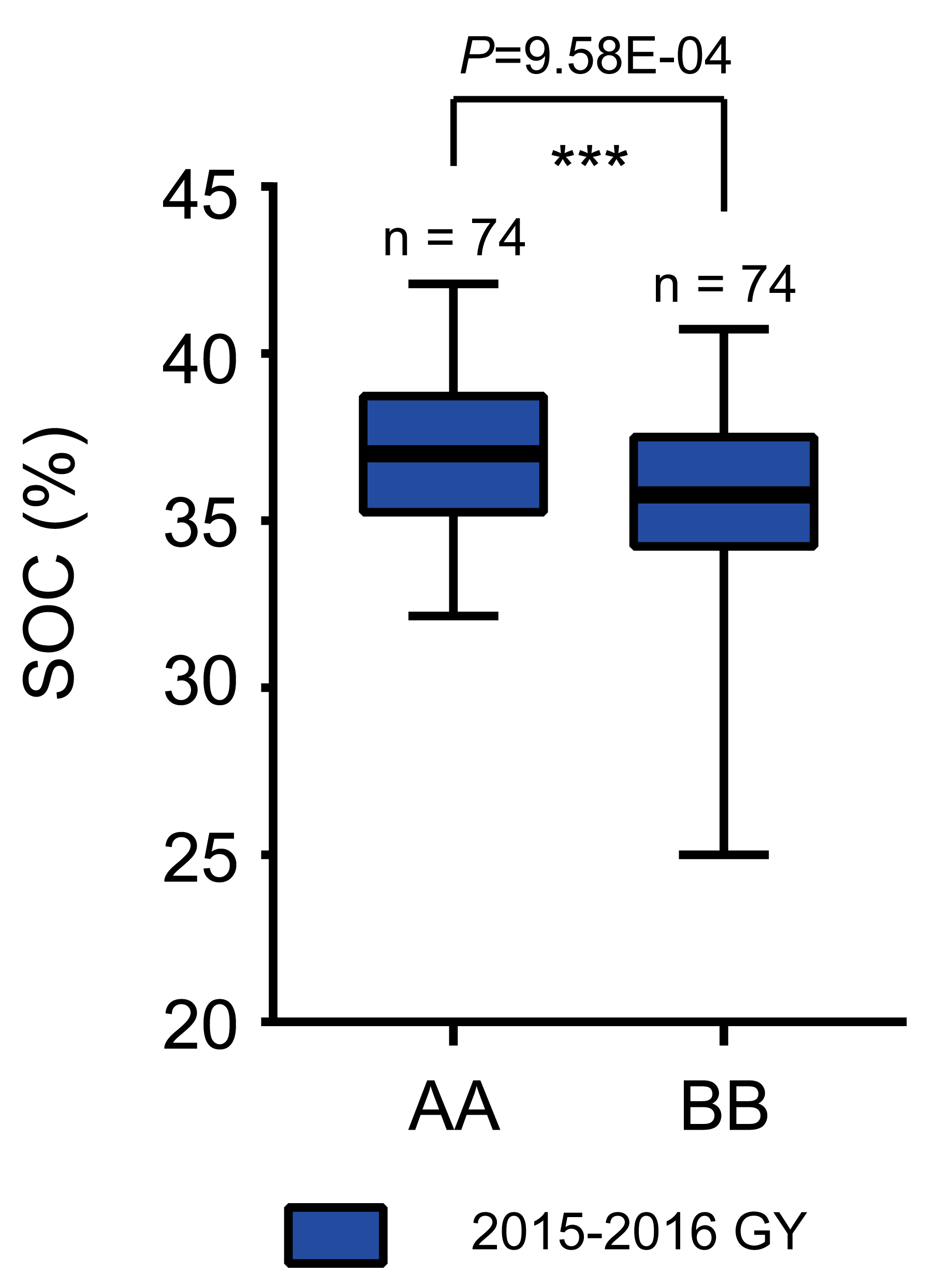

Supplement: Supplementary Figure 6 — The effect analysis of the major QTL for SOC in 2015–2016 GY. AA and BB was the genotype of each flanking markers linked the major QTL in the two parents. The ‘n’ represented the number lines in the RIL population conferring the corresponding genotype. *** represented significance level of the Student’s t-test, p < 0.001. [file Image_6.TIF]

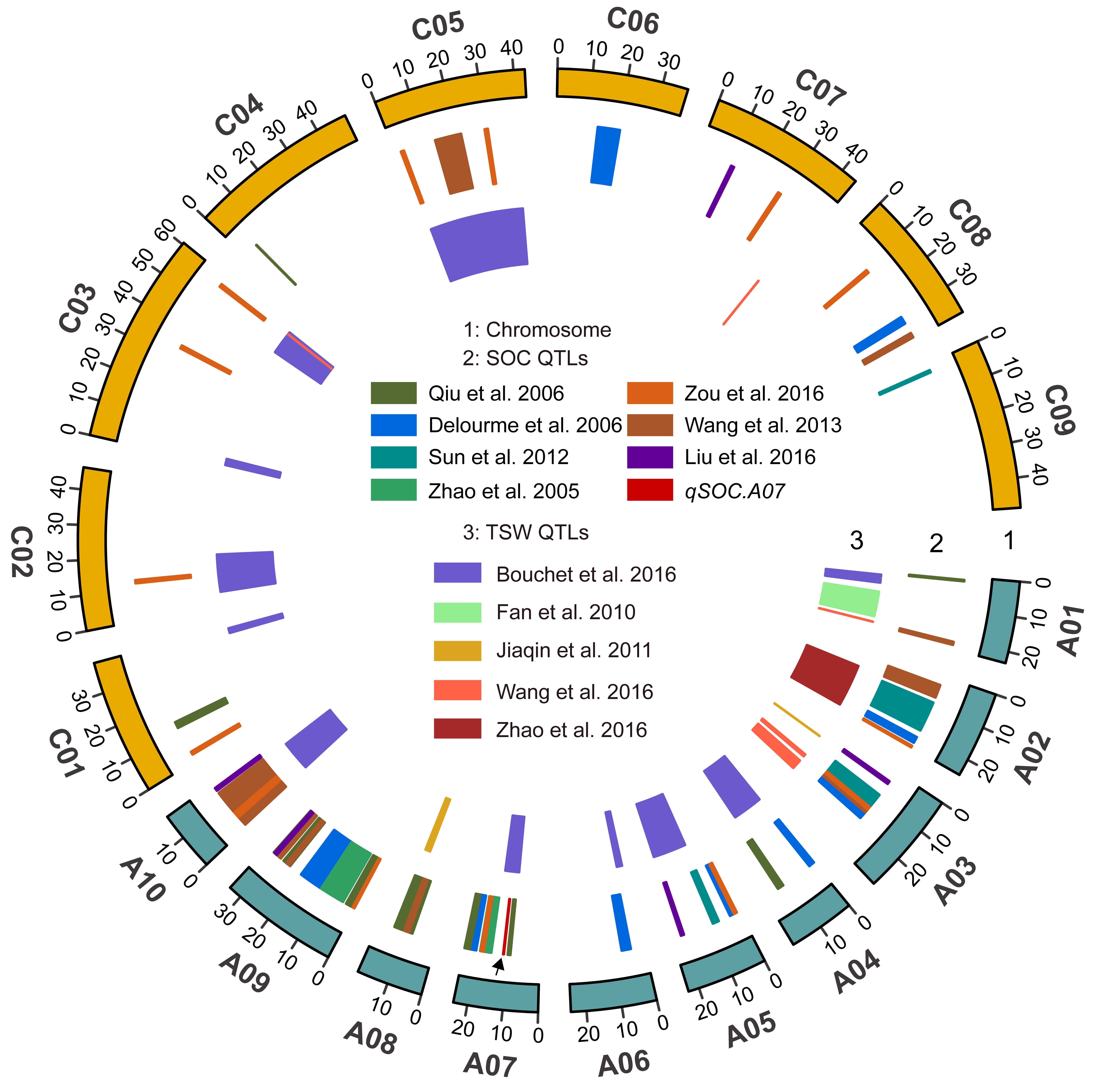

Supplement: Supplementary Figure 7 — QTLs for TSW and SOC identification in previous studies. [file Image_7.TIF]
